# Convalescent Plasma against COVID-19: A Broad-Spectrum Therapeutic Approach for Emerging Infectious Diseases

**DOI:** 10.3390/microorganisms8111733

**Published:** 2020-11-05

**Authors:** Marijn Thijssen, Timothy Devos, Hanieh-Sadat Ejtahed, Samad Amini-Bavil-Olyaee, Ali Akbar Pourfathollah, Mahmoud Reza Pourkarim

**Affiliations:** 1 Laboratory for Clinical and Epidemiological Virology, Department of Microbiology, Immunology and Transplantation, Rega Institute for Medical Research, KU Leuven, 3000 Leuven, Belgium; marijn.thijssen@kuleuven.be; 2 Department of Haematology, University Hospitals Leuven, 3000 Leuven, Belgium; timothy.devos@uzleuven.be; 3 Obesity and Eating Habits Research Centre, Endocrinology and Metabolism Clinical Sciences Institute, Tehran University of Medical Sciences, Tehran 1411413137, Iran; ejtahed-h@sina.tums.ac.ir; 4 Endocrinology and Metabolism Research Centre, Endocrinology and Metabolism Clinical Sciences Institute, Tehran University of Medical Sciences, Tehran 1411413137, Iran; 5 Biosafety Development Group, Cellular Sciences Department, Amgen Inc., One Amgen Center Drive, Thousand Oaks, CA 91320, USA; samad@amgen.com; 6 Department of Immunology, Faculty of Medical Sciences, Tarbiat Modares University, P.O. Box 14115-111, Tehran 14117-13116, Iran; pourfa@modares.ac.ir; 7 Health Policy Research Centre, Institute of Health, Shiraz University of Medical Sciences, P.O. Box 71348-45794, Shiraz 71348-54794, Iran; 8 Blood Transfusion Research Centre, High Institute for Research and Education in Transfusion Medicine, Tehran 14665-1157, Iran

**Keywords:** antiviral, convalescent plasma, neutralizing antibodies, COVID-19, SARS-CoV-2, pandemic, preparedness, blood

## Abstract

In the lack of an effective vaccine and antiviral treatment, convalescent plasma (CP) has been a promising therapeutic approach in past pandemics. Accumulating evidence in the current severe acute respiratory syndrome coronavirus 2 (SARS-CoV-2) pandemic corroborates the safety of CP therapy and preliminary data underline the potential efficacy. Recently, the Food and Drug Administration (FDA) permitted CP therapy for coronavirus disease 2019 (COVID-19) patients under the emergency use authorization, albeit additional clinical studies are still needed. The imminent threat of a second or even multiple waves of COVID-19 has compelled health authorities to delineate and calibrate a feasible preparedness algorithm for deploying CP as an immediate therapeutic intervention. The success of preparedness programs depends on the interdisciplinary actions of multiple actors in politics, science, and healthcare. In this review, we evaluate the current status of CP therapy for COVID-19 patients and address the challenges that confront the implementation of CP. Finally, we propose a pandemic preparedness framework for future waves of the COVID-19 pandemic and unknown pathogen outbreaks.

## 1. Introduction

The world is currently confronted with an unprecedented threat of a novel coronavirus called severe acute respiratory syndrome coronavirus 2 (SARS-CoV-2) [1]. In late December 2019, China reported a cluster of pneumonia cases of an unknown cause in Wuhan, Hubei Province [2]. In early January 2020, SARS-CoV-2 was reported as the causative pathogen for the majority of pneumonia cases [3]. From Wuhan, the virus quickly spread across the globe and urged the World Health Organization (WHO) to declare a pandemic in March 2020 [4]. As of 29 October, 2020, 45,423,699 people have been diagnosed with the infection and 1,187,529 died worldwide. By applying a series of multidisciplinary approaches, all countries are trying to timely diagnose infected individuals to prevent the spread of the virus and its associated disease [5]. Current available therapeutic measures primarily entail supportive care, e.g., oxygen ventilation, combined with the administration of anti-inflammatory drugs [6]. Recently, the off-label use of antivirals, like the anti-influenza drug favipiravir and anti-Ebola virus drug remdesivir, is evaluated for safety and efficacy against SARS-CoV-2. Until now, only remdesivir has been approved under the emergency use authorization (EUA) by the Food and Drug Administration (FDA) for coronavirus disease 2019 (COVID-19) patients after reporting a reduced time to recovery in treated patients [7,8]. However, recent clinical data also questions the use of remdesivir [9], which exemplifies the difficulties the scientific world is currently facing to find and evaluate effective treatment options for COVID-19 patients. Considering the rapid spread and lack of specific treatments, it seems that humankind is not well equipped to combat this deadly virus. However, the history of medicine teaches us the availability of a widely used strategy that has been applied in previous epidemics called passive immunity.

Passive immunity is a relatively old approach that dates back to 1890, decades before the development of antimicrobial therapies [10,11,12]. Emil Behring was the first to apply this approach against diphtheria and tetanus, for which he received the first Nobel Prize in Medicine in 1901 [13]. Passive immunity comprises the collection of plasma from recovered individuals and the transfusion of this plasma to infected patients. The therapeutic effect is prompted by specific antibodies, i.e., neutralizing antibodies, directed against toxins or antigens of the pathogen [14]. After its initial use, convalescent plasma (CP) therapy was introduced and applied in postexposure prophylaxis of several infectious diseases like rabies, polio, measles and hepatitis. The first mass usage of CP therapy was reported during the influenza pandemic in 1918 and saved thousands of lives [15,16].

CP could be considered an effective therapy in emergency situations where specific treatments are still lacking. CP was used in recent disease outbreaks and pandemics. For instance, during the outbreak of SARS in 2003 [17], the influenza A H1N1 pandemic in 2009 [18] and the outbreak of MERS in 2015 [19], passive immunization through CP therapy demonstrated its curative potential. Furthermore, the WHO recommended the use of CP collected from Ebola virus disease survivors to treat newly infected patients in 2014 [20,21]. The application of CP or immunoglobulins (purified antibodies from CP) in these settings significantly improved clinical parameters by modulating immune responses (e.g., levels of IL-6 and IL-10) and drastically reducing viral load, which resulted in a shortened hospital stay and decreased mortality rate [22,23,24,25,26,27]. These findings demonstrate that CP therapy holds great potential for treating emerging infectious diseases in the lack of specific treatment strategies.

CP therapy could potentially control and divert the epidemic trend and save human lives. This treatment can be adopted in emergency situations to prevent the excessive flux of cases from overcrowded hospitals to intensive care units (ICUs). Besides CP, the pharmaceutical industry has expressed its interest in passive immunity by embarking on the search for effective monoclonal antibodies (mAbs). However, in contrast to mAbs, CP therapy requires fewer resources and can be quickly implemented in an efficient and safe manner. Therefore, this strategy could be added to a pandemic preparedness roadmap, in which the involved organizations in CP clinical trials are rapidly informed and organized once emerging infectious diseases appear.

With a daily increase in the number of new SARS-CoV-2-infected cases and existing concerns for multiple waves of the pandemic in the coming seasons, it is currently the right time to outline the challenges that confront the implementation of CP in clinical practice. On 23 August 2020, the FDA issued a EUA for convalescent plasma in COVID-19 patients. Importantly, this authorization should not replace clinical trials since critical data concerning safety and efficacy are still needed [28,29,30,31]. Furthermore, a variety of known and possible unknown practical aspects should be evaluated and documented in CP therapy guidelines. A roadmap can be tailored and adopted in a preparedness strategy for the next waves of the COVID-19 pandemic and future disease outbreaks. This preparedness framework for deploying CP therapy against emerging infectious diseases could be implemented in a standardized early-response strategy. The prompt implementation of this potentially curative treatment includes different steps that have individual challenges. Based on the most recent literature, we have evaluated the ongoing deployment steps of CP and current clinical trials to monitor crucial challenges. We strongly believe that it is currently the time to learn and take initiatives to adopt this approach in a standardized preparedness protocol.

## 2. Current Challenges for CP Therapy

### 2.1. Timing of CP Therapy Implementation

After identifying the novel coronavirus as the etiology of COVID-19 in China, countries started to look for antiviral therapies and the development of potential vaccines. Simultaneously, experts expected to obtain early FDA approval to launch clinical trials to evaluate the safety and efficacy of CP therapy. However, the initial strategy presented by the WHO primarily focused on preventing the spread of the virus [32]. In contrast, China, being the first country that was severely affected by COVID-19, started to evaluate CP treatment in a preliminary uncontrolled case series including five critically ill patients with COVID-19 [33]. After receiving positive results, additional cases were added to these trials [34,35]. Remarkably, it took a long time for the FDA to seriously consider CP therapy and approve clinical trials. Only until COVID-19 slammed New York City, Governor Andrew Cuomo announced the application of CP therapy for critically ill patients [36]. According to the FDA guidelines, clinicians and investigators who request the use of convalescent plasma are required to apply for an emergency investigational new drug procedure. Under this regulation, eligible patients for CP therapy should be diagnosed with “serious or immediate life-threatening COVID-19 infections.” This requirement excludes the prophylactic use of CP therapy. The FDA has defined a specific set of parameters to diagnose “severe disease”, including dyspnea, a respiratory frequency of ≥30 breaths per minute, a blood oxygen saturation of ≤93%, a ratio of partial pressure arterial oxygen to fraction-inspired oxygen (PaO2/FiO2) of <300 or lung infiltrates of >50% within 24 to 48 h. Additionally, “life-threatening disease” is defined by either respiratory failure, septic shock or multiple organ dysfunction [37].

Next to the FDA guidelines, the European Medicines Agency (EMA) also implemented a protocol for starting clinical trials to evaluate CP therapy in Europe [38]. The American Association of Blood Bank (AABB), as well as European blood centers, e.g., the European Blood Alliance (EBA) and the Red Cross and non-European countries, have launched websites to recruit COVID-19 survivors who wish to donate plasma [39].

Concerted efforts primarily aim to ramp up the collection and transfusion of CP to treat COVID-19 patients and curb the pandemic trend. In hindsight, health experts witnessed a substantial delay in the announcement of CP clinical trials by health authorities at an international level, while swift decision-making is essential for the wide implementation of CP therapy under emergency conditions [40]. Ideally, a pandemic preparedness roadmap could guide and expedite the clinical evaluations of CP therapy as an urgent aid. By streamlining clinical studies and providing rapid communication channels, promising treatments like CP therapy could avoid the progress of disease and ultimately might reduce mortality rates.

### 2.2. Source of Convalescent Plasma

Unlike chemically based medicine, CP is a biological therapy that relies on the availability of patients that recovered from the infection. Eligible patients for plasma donation should have raised substantial titers of neutralizing antibodies, which is pivotal for the success of this treatment. The recruitment of plasma donors starts with calling the recovered patients by hospitals, family doctors or the public announcement of blood transfusion organizations like the Red Cross. Furthermore, healthcare personnel can inform the recovered patients before discharge to encourage them to donate plasma. Throughout the recruitment process, the privacy and confidentiality of the donor should be guaranteed and comply with ethical regulations (e.g., informed consent). Donated plasma should be collected through voluntary and nonremunerated transfusions. Directed donations are not recommended and plasma units should be sent anonymously to the hospitals [16]. Currently, the FDA recommends collecting CP at least 28 days after resolving COVID-19 symptoms or after 14 days in combination with two negative molecular tests (with at least a 24 h interval). These timeframes are chosen to ensure the eradication of the virus and the development of neutralizing antibodies [41,42,43,44].

To construct a timeline for CP collection, it is highly important to understand the neutralizing antibodies’ kinetics postinfection and recovery. For instance, knowing when the antibodies reach the maximum concentration after infection and for how long it persists in the blood is pivotal information. Results showed that in SARS-CoV-infected cases in 2003, the neutralizing antibody concentration reached the highest level within four months after the onset of disease and remained positive up to 24 months postinfection [45,46]. In contrast, in MERS-CoV-infected patients, the antibodies declined already three months after the disease [19]. For SARS-CoV-2, it is currently too soon to determine the durability of the neutralizing antibodies. However, the available data suggest that these antibodies appear approximately nine days after the initial infection [47]. Another study reported a 50% to 100% seroconversion 7 and 14 days after the onset of infection, respectively [48].

It has been reported that some recovered patients do not develop a detectable titer of neutralizing antibodies [49]. Similar cases have been observed in the Ebola virus, influenza and SARS-CoV-1- and SARS-CoV-2 infected patients [50,51,52,53]. The level of antibody production has often been linked to the severity of the disease. For instance, asymptomatic cases appear to secrete lower titers of IgG and neutralizing antibodies, which eventually drop after convalescence [54]. The underlying reason for this difference could be related to the immunogenetic background of the individual and the engagement of cellular immunity instead of the humoral immune system [49,55].

The success of collecting convalescent plasma depends on the willingness and eligibility of the recovered individual to donate plasma. According to previous experiences, some recovered patients avoid participating in the collection of CP. Furthermore, the donor eligibility to donate plasma is based on health records, blood screening, hematocrit level and platelet count according to the BRN standards of the WHO [44,56,57]. According to previous data, almost one-third of recalled convalescent individuals donate plasma [52]. During an epidemic, the emergency need for CP could reach exceptional heights and overload the blood donation infrastructure. This imposes new challenges in coordinating the supply of convalescent plasma for treating infected patients and routine services of blood centers. It seems that plasma donor availability and adequate logistic support are both inseparable steps for CP therapy.

### 2.3. Precautionary Requirements for Plasma Donors

Precautionary strategies should be implemented for the safe administration of CP therapy to mitigate the risk of transmitting SARS-CoV-2. Regarding the reports of SARS-CoV-2-positive blood samples, implementing RT-PCR screening tests for the donor and convalescent plasma before transfusion is highly recommended. This test could be listed in the standardized blood screening panel that is used to determine donor eligibility for blood donation. Without the approval of the blood screening department, collected CP should be kept in quarantine (within 24 h after collection) and can only be released for transfusion after receiving clearance.

The implementation of pathogen-reduction techniques provides an extra safety step in preventing the transmission of SARS-CoV-2 through blood transfusion. However, these techniques are not available in all countries. Therefore, SARS-CoV-2 molecular screening of the donated blood is recommended to guarantee the safety of the plasma. Besides molecular techniques, antibody testing has been proposed to confirm viral clearance in the donor. This approach can be faster and less laborious, albeit antibodies are not good indicators of viral clearance [58]. For instance, seroconversion is usually time-dependent with a delay in the appearance of antibodies compared to viral RNA/DNA. Therefore, the safety and sensitivity profile of molecular-based assays makes it the preferred diagnostic approach [59,60,61]. However, recent findings suggest a combination of both RNA and antibody tests to improve patient diagnosis and prognosis [58]. The importance of a combined approach is highlighted when a decision for treatment should be made on short notice [62]. Finally, novel approaches have been developed (e.g., rapid antigen tests) and might be implemented in diagnostic guidelines in the near future [63].

Some reports have demonstrated detectable levels of SARS-CoV-2 RNA in patients discharged from hospitals [64,65]. In these cases, the safe use of CP and the safety of people in their environment could be compromised [64]. Furthermore, inconsistent test results between different biological specimens add to the existing concerns over patient infectiousness. Generally, oral swabs are used for the molecular diagnosis of SARS-CoV-2. However, recent investigations showed that molecular tests for anal swabs were positive, while oral samples were negative. Furthermore, in some patients with diagnosed blood viremia, both oral and anal swab were negative [35]. These findings and other reports raise concerns over possible false-negative results of the nasopharyngeal swab PCR test [62,66,67]. Furthermore, inconsistent results could be related to personal skill, time of sampling and the performance of the RT-PCR assay [58].

Conflicting results are concerns in routine screening and could have a far-reaching impact on CP collection. The clinical relevance of finding positive stool samples combined with negative oral swabs should be further investigated. Although the symptoms could be resolved, the patients could still carry the virus while visiting the plasma donation center [16,35]. Recent publications demonstrated that 30% of patients reported gastrointestinal symptoms, e.g., diarrhea, and the presence of the virus in their stool [68]. The cellular tropism of SARS-CoV-2 largely depends on the expression of the ACE2 receptor on the cell surface, which is widely abundant in intestinal cells. Furthermore, shedding of SARS-CoV-2 in the urine has also been reported [69]. Notably, asymptomatic infected cases might have a longer duration of viral shedding than symptomatic patients, albeit the clinical relevance of this difference should still be determined [54]. These findings clearly indicate that precautionary guidelines are highly needed in handling possible infectious material.

### 2.4. Plasma Donation and Postdonation Challenges

The collection of plasma should comply with the highest quality standards to guarantee the safety of both the donor and recipient. Preferably, plasma should be collected through a routine apheresis method. This method is an automated and closed system that separates 400–800 milliliters of plasma from whole blood and returns the remaining cells and other blood components to the donor [16]. The total volume of collected plasma can be divided into multiple units. Plasma can also be extracted from whole blood donations, albeit this could result in a variable volume of plasma and an unnecessary loss of red blood cells [44]. Furthermore, this method restricts the frequency of repeated donations in a short period of time [41]. It is necessary to consider at least one or eight weeks between the following plasma or whole blood donation, respectively. The interval between consecutive donations depends on country-specific guidelines and the physical condition of the donor [44]. In all cases, compatibility of the ABO blood group between donors and recipients should be regarded [33].

In addition to a high-quality plasma collection infrastructure, the viral neutralizing capacity of CP should be determined postdonation. The titer of neutralizing antibodies can be highly different between individuals and might not reach sufficient thresholds [16,70]. The potency of CP therapy largely depends on the neutralizing capacity. Therefore, it is highly recommended to determine the neutralizing antibody titer in the donated plasma [41]. Surprisingly, the titration of antibodies was fully ignored in some studies, which could jeopardize the validity of clinical outcomes [29,64]. Previous attempts of treating Ebola-virus-infected patients with CP containing low-antibody titers were unsuccessful, even though two consecutive units had been transfused [71]. To assess the neutralizing capacity of CP, the plaque reduction neutralization test (PRNT) is currently the golden standard [48]. This test requires the exposure of cell lines to SARS-CoV-2 viruses. With a minimal duration of five days, this method is relatively time-consuming [33,34]. In addition to PRNT, pseudotyped assays, including vesicular stomatitis virus and lentivirus, are currently available. Both methods rely on the exposure of viable viruses to target cells and should be performed in high-biosafety-level laboratories. Recent advances in surrogate virus neutralization tests implemented ELISA principles to measure the neutralization capacity of antibodies. These methods seem promising for applications outside high-biosafety-level environments, albeit their use should be further assessed for SARS-CoV-2 [72].

Currently, some diagnostic and quantification methods, e.g., ELISA, are making progress to replace PRNT [16,31,51,58,70,73]. ELISA assays primarily detect the total antispike IgG antibodies, while PRNT determines the total neutralizing antibodies [16]. This marks important differences between both approaches, since not all antibodies can neutralize the virus, which could result in an overestimated efficacy based on ELISA tests [74,75]. However, recent reports of a strong correlation between antispike antibodies and neutralizing antibodies against SARS-CoV-2 favor the potential application of ELISA [51,76]. Beyond detecting effective titers of antibodies in CP, reliable serological assays are important assets to evaluate the immune response in SARS-CoV-2-infected patients. In light of the recent emerging data concerning reinfected cases [77], understanding antibody kinetics is imperative for future containment strategies and vaccine development. To implement an additional safety threshold, some ELISA assays recommend preheating the serum/plasma to inactivate residual viruses. Finally, the applied method should have high specificity for SARS-CoV-2 to avoid cross-reactions with human coronaviruses that cause seasonal common cold (alpha coronaviruses (NL63, 229E), beta coronaviruses (OC43 and HKU1)) [78].

A standardized level of neutralizing antibodies is still lacking. Early clinical case series with a limited number of patients used various neutralizing antibody concentrations. In China, an initial study started with a neutralizing antibody titer of >1:40, while another trial observed an improved efficacy of CP therapy with more than 1:640 [33,34] (Table 1). In the following studies, variable titers of neutralizing antibodies were used ranging from 1:20 to 1:1280 [79,80,81]. Besides titrating neutralizing antibodies by PRNT, multiple trials measured the SARS-CoV-2 antispike antibodies with ELISA techniques. Here, an antispike IgG titer of >1:320 was mostly used [51,70,73,82]. A recent study showed that a concentration of 1:1280 for IgG antibodies against the spike-receptor-binding domain was equal to a neutralizing antibody titer of 1:80 [30]. However, there are a few studies that have considered a titer below this threshold (>1:160) [30,83]. Since the level of antispike antibodies between donors can be highly variable, some trials applied a range of antibody concentrations. For instance, titers of 1:160–1:1280 or 1:150–1:1350 have been used [30,31,84]. In addition, some studies only reported the presence IgG without determining the antibody levels [85,86], while others only used the signal-to-cut-off value of the ELISA test to confirm antispike IgG positivity [87]. Current clinical evidence emphasizes that the neutralizing antibody titer plays a critical role in the potential efficacy of CP in COVID-19 patients. However, there is still a disparate usage of various methods and corresponding antibody levels in current reports. Therefore, we strongly recommend using a standardized method and neutralizing antibody titers to evaluate the applicability of CP therapy in future clinical trials.

### 2.5. Timing of CP Transfusion

In addition to neutralizing antibody titers, a growing body of evidence implies that the timing of CP therapy is an important indicator of therapy success. Previous reports on CP therapy revealed that a delayed administration could be one of the main causes of therapy failure [71]. Until now, studies have applied a different timing of CP administration, ranging from 1 to 50 days posthospitalization (Table 1). The effectiveness of neutralizing antibodies for COVID-19 decline in advanced stages of the disease, which are characterized by uncontrolled inflammatory responses [36,88]. In these stages, therapy should divert to medicines that act on the underlying immune pathology. However, some studies suggest that, in these groups of patients, CP therapy could elicit clinical improvements and promote viral clearance [30,64,88]. Clinical trials have demonstrated that recovery markers were more pronounced in patients that received CP therapy within 14 days after the onset of illness compared to later time points [33,34]. The highest clinical impact has been observed at the beginning of the symptomatic phase or as a postexposure prophylactic measure [16]. These observations accommodate observations in other respiratory infections [30,64,70]. Recent data suggest that administering CP therapy later than 10 days after hospitalization does not seem to improve clinical signs of the patients [81]. The absence of clinical effects could be attributed to the presence of pretreatment neutralizing antibodies in the patients. In contrast, a significant reduction of mortality was observed when CP was administered within 72 h after hospitalization and with a high antispike protein receptor-binding domain titer of >1:1350 [31,87].

The most optimal timing for CP therapy is expected to be within three to five days after the onset of disease symptoms. Within this timeframe, the antiviral activity of CP can prevent the development of tissue damage [88,89]. The optimal timing largely depends on the viral dynamics of SARS-CoV-2. Preliminary data revealed that the viral load peaked during the first week of infection and a primary immune response developed after 10 to 14 days [38]. Accordingly, CP therapy was more effective when administered shortly after infection by suppressing SARS-CoV-2 viremia [30,38,88]. In this context, CP therapy can mitigate disease progression and reduce the flow of patients to the ICU [90,91]. Therefore, it is strongly advised to administer CP therapy close to the moment infection in the absence of baseline neutralizing antibodies in the patient.

### 2.6. Dosage of CP Therapy

The dosage of CP can be highly variable and depends on clinical indications, either preventive or curative. The required dosage or volume of CP is primarily determined by the neutralizing antibodies’ titer, estimated half-life of the antibodies and body weight of the recipient [26]. The volume of plasma that should be administered per kg of body weight depends on the antibody titer. For instance, 5 mL/kg is of plasma is required with an antibody titer of ≥1:160. To unify a national or international preparedness program, a standardized antibody concentration is highly recommended for preparing plasma units. In previous epidemics/pandemics, e.g., Influenza H1N1, SARS and MERS, the administration of a single dose of CP has been used [18,92,93]. The current literature indicates that studies followed different procedures in terms of CP dosage for COVID-19 patients (Table 1). One study prescribed two doses of 200–250 mL with an antibody titer of 1:40 [33], while in another trial, one dose of 200 mL was used with a neutralizing antibody titer of 1:640 [34]. To increase the level of protection, two or three dosages of 200 mL plasma are recommended [44]. Furthermore, plasma from two different donors can be used to acquire diverse fractions of antibodies that could provide a therapeutic benefit. Besides multiple dosages, a single unit with a higher volume (e.g., 300 mL of CP) can also be administered [31]. However, to implement a universal recommendation, future strategies should define a uniform dosage that is preferably expressed in the required neutralizing antibody titer per kilogram of body weight. In clinical practice, CP therapy will be combined with standard treatments of antivirals and/or corticosteroids [33,34]. In this context, it is important to determine the clinical situation of the patient in making decisions over the administration of additional units. For instance, improvements in clinical parameters, e.g., O_2_ saturation level, could indicate if the transfusion of more units is needed [38].

### 2.7. Post-CP Transfusion Follow-Up

The process of CP transfusion should be closely monitored. Generally, the transfusion of a single dose takes approximately 30 min to 1 h [64]. Some procedures use a variable speed of administration starting with 10 mL in the first quarter up to 100 mL per hour [30]. During the administration of CP, healthcare personnel should closely monitor the clinical status of the recipient by checking every 15 min [64]. During the first 4–7 h post-transfusion, the impact of the therapy should be frequently evaluated until hospital discharge [29]. The efficacy will be determined based on the collected data at different stages of the treatment.

According to recent clinical trials, the impact of CP therapy on clinical parameters should appear during the first week after transfusion [33,34,70]. Multiple clinical and paraclinical parameters can be evaluated to follow the clinical status of the patients at different stages of the treatment. Monitoring clinical parameters such as cough, fever, oxyhemoglobin saturation, extracorporeal membrane oxygenation, sequential organ failure, resolution of pulmonary lesions and/or the resolution of ground-glass opacities pre- and post-transfusion is highly recommended. Paraclinical markers like D-dimers, coagulation, inflammatory factors (e.g., C-reactive protein), lymphocyte count, procalcitonin, IL-6, serum antibody titer (IgG, IgM, and neutralizing antibodies) and a biochemistry panel of liver and kidney function require careful follow-up [33,34,64,73,94]. Since the fatality rate is closely related to hypercytokinemia and viral load, these two markers should be closely monitored.

To evaluate the efficacy of CP, it is important to measure the level of neutralizing antibodies before and after CP therapy by longitudinal sample collection. In two recent studies performed in China, a significant increase in neutralizing antibodies of more than 1:640 [34] and >1:480 [33] was observed after CP transfusion. Both studies reported an improvement in patient clinical status, including an increase in oxyhemoglobin saturation and neutralization of viremia, after more than one-week post-transfusion. Furthermore, a negative viral load was observed within 1–12 days post-therapy [33,34,73]. Viral clearance can be monitored and used as a recovery marker that should appear between 24 to 72 h after the transfusion of CP [30].

### 2.8. Risks

Current data indicate that the occurrence of serious adverse events (SAEs) after CP therapy is very low and mostly absent [29,30,31]. However, as for many treatments, there are several risk factors that might endanger the success of CP transfusion. A recent study on 5000 patients with COVID-19 treated with CP showed that 0.04% experienced SAEs attributed to the therapy (2 cases per 5000). The patients receiving CP were hospitalized in the COVID-19 units or admitted in the ICU and reported a mortality rate of 15–20% [95,96] and 57% [96], respectively. Patients in the ICU were more often diagnosed with comorbidities and organ failure [95]. In these cases, CP was prescribed as salvage therapy.

Transfusion of blood products carries a risk of transmitting bloodborne pathogens. Several strategies have been implemented to minimize this risk, including the screening of donated blood and a medical background assessment of the donor. Besides screening, blood products can be actively treated with pathogen-reduction techniques [43]. However, these procedures have not been advised in the authorized FDA protocol [37]. Pathogen-reduction technologies can decrease the risk of transmitting bloodborne infections and possible superinfection with SARS-CoV-2 [44]. Various pathogen-reduction methods can be deployed to inactivate potential viral pathogens in donated plasma, including methylene blue photochemistry, ultraviolet C light or riboflavin and ultraviolet light [52,97,98,99,100]. Importantly, these additional steps should not lead to a decrease in neutralizing antibodies against SARS-CoV-2 [53]. Although SARS-CoV-2 RNA has been detected in the plasma, there is currently no evidence of transmission through blood/plasma transfusion [100,101,102].

Other risks might emerge from transfusion-related reactions, including transfusion-related acute lung injury (TRALI) and transfusion-associated circulatory overload (TACO). These two SAEs can lead to pulmonary edema and are potentially lethal [103]. The chance of developing TRALI is 1 per 5000 transfusions and has been reported in Ebola-virus-infected patients who received CP therapy [104,105]. TRALI is caused by anti-HLA antibodies that can emerge during pregnancy. Therefore, plasma is preferably collected from women who have never experienced pregnancy; otherwise, the plasma should be checked for anti-HLA antibodies [44]. TRALI occurs a couple of hours after transfusion and can have a severe clinical course. In addition to TRALI, TACO is caused by fluid volume overload and is specifically harmful to patients suffering from heart and kidney diseases [29,70]. The risk of TACO can be significantly reduced by closely monitoring the transfused volume. Furthermore, coagulopathy is also recognized as a transfusion-related disorder that is caused by the presence of coagulating factors in the plasma [90,106]. The occurrence of these blood transfusion complications emphasizes the importance of a careful patient follow-up after administering CP treatment.

Another risk factor of CP is related to the presence of non-neutralizing or suboptimal antibodies. Docking of these antibodies on the surface of the virus could potentiate the cellular uptake of the virus in a mechanism called antibody-dependent enhancement (ADE) [107]. Instead of neutralizing the virus, this process might exacerbate clinical severity and worsen the disease outcome when CP is administered. ADE has been reported in previous coronavirus infections (e.g., SARS-CoV and MERS) as well as dengue viruses and HIV [108,109,110,111,112]. Until now, none of the recent CP clinical trials in COVID-19 patients have reported signs of ADE post-transfusion [33,34,73]. When CP is used as a prophylactic treatment, the possible occurrence of ADE warrants more attention [16]. In addition to ADE, it is hypothesized that the transfusion of CP might inhibit the activation of the adaptive immune system, which could impede the development of immunity and possibly long-term protection. However, current data on this hypothesis are still inconclusive and need more evidence [70,113].

### 2.9. CP Technology: Hyperimmunoglobulin and Monoclonal Antibodies

To implement an efficient and safe infrastructure for CP therapy during a pandemic, the recruitment of donors and mass collection of plasma are necessary within a short timeframe. This requires access to specific technologies for blood transfusion and blood fractioning. Recent improvements in plasma apheresis techniques (e.g., systems that separate plasma and return the remaining cells to the donor) shortened the time and improved the efficiency of plasma. Depending on the available facilities and the phase of the pandemic, collected plasma could be fractionated to acquire hyperimmunoglobulin (HIG) formulas [53]. The benefit of using HIG formulation is that the titer, affinity and specificity of the antibodies in each dose can be accurately determined and standardized before transfusion [89]. Furthermore, the use of HIG does not require blood group compatibility. Biopharmaceutical companies have expressed their interest in being involved in clinical trials evaluating HIG therapies [39,89]. However, there are some crucial challenges that should be addressed before HIG therapy can be applied in clinical practice. For instance, distribution channels of HIG formulas require robust logistic infrastructures [52]. The production time of HIG formulas can reach up to six months [114], which limits the use during emergency situations at the very onset of a pandemic. Moreover, the limited availability of plasma and fractionation systems to prepare immunoglobulins are critical hurdles for the production of HIG in resource-limited countries [16]. Therefore, CP therapy is a more accessible option for offering a rapid solution in the frontline of a pandemic. Furthermore, stocking frozen convalescent plasma units could be a key point of preparedness strategies for the next waves of the pandemic [53].

Another prophylactic or treatment option includes the use of mAbs derived from B lymphocytes. Like antibodies, these cells can be isolated from individuals that survived viral infection. This approach has demonstrated promising effects in animals infected with SARS-CoV-1, in vitro models of MERS-CoV and patients infected with the Ebola virus [115,116,117,118]. The efficacy of mAbs therapy is currently being investigated for COVID-19 indications. Recently, this therapy approach has drawn attention following the treatment of the President of the United States with a promising experimental cocktail of two humanized mAbs manufactured by Regeneron [119]. In addition to Regeneron, multiple stakeholders joined the search for effective antibodies against SARS-CoV-2. In April, Vir Biotechnology reported the SARS-CoV-2-neutralizing capacity of antibodies recovered from SARS-CoV survivors, which target a highly conserved region in both coronavirus species [120]. The in vitro production of mAbs circumvents the drawbacks of CP therapy that include therapy standardization and variability in neutralizing capacity. Recent preliminary data demonstrated promising efficacy and safety of mAbs treatment in COVID-19 patients [120]. However, large-scale clinical trials are needed to further deepen our knowledge concerning the possible risks and uncertainties of these treatments [121]. Although mAbs are potent compounds for COVID-19 patients, the development and availability of this treatment require advanced technologies and funding. For instance, the isolation and cloning of specific B cells that produce neutralizing antibodies is a time-consuming and difficult process [122,123,124]. These aspects could be an obstacle for use in resource-limited countries. The time needed for the development and dissemination of mAbs formulas could be a limiting factor in emergency situations. Therefore, we expect CP to outpace the applicability of mAbs treatment in the initial phases of a pandemic caused by a novel pathogen. CP therapy could take advantage of the established blood transfusion infrastructure. In the long term, we expect that potent mAbs therapies will replace CP therapy, albeit emergency situations call for rapid treatment options that are readily available [5,30,88]. Therefore, clear guidelines on how the safety and efficacy of CP therapy should be evaluated in future pandemics are highly needed for the implementation of this treatment.

## 3. Implementation of CP Therapy in Pandemic Preparedness

**Figure 1:**
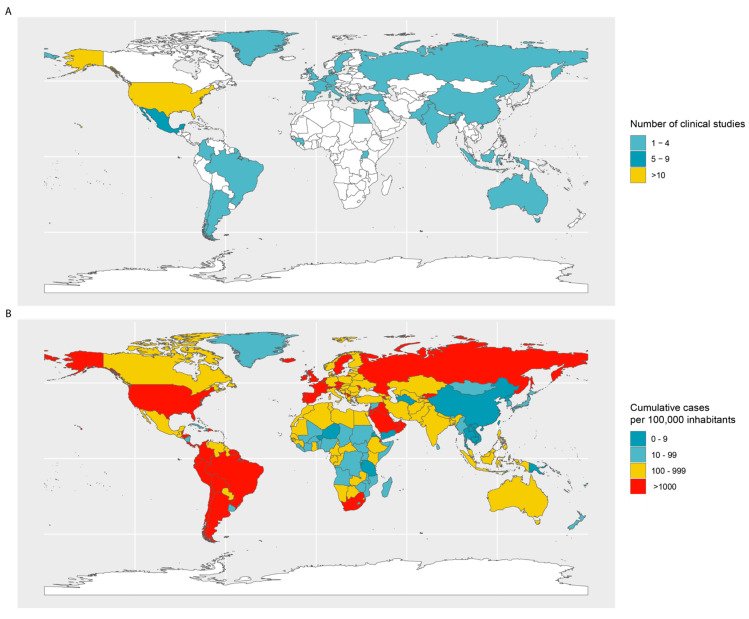
(**A**) Global distribution of the registered clinical trials for convalescent plasma therapy. (**B**) Dispersal of the number of cases per country as of 29 October 2020 (cumulative cases per 100,000).

Convalescent plasma therapy is an old therapeutic tool that, after the introduction of antibiotics, was pushed to the side-line. However, the threat of emerging pathogens in past decades revived the use of CP therapy while immediate vaccines and therapeutic medicines were still lacking. Today, the world is confronted with a devastating pandemic. Now more than ever, CP therapy is in the spotlight again. CP therapy has demonstrated high potential as a possible curative intervention and could curb infectious pandemics. The characteristics of this treatment make it an appropriate and prompt therapeutic strategy that can be implemented in pandemic preparedness. Therefore, clinical studies have been launched in all continents, with the majority of trials registered in the Americas and Europe (Figure 1A). Although COVID-19 is spread across all continents, there is still a large discrepancy of CP clinical trial coverage between developing and developed countries (Figure 1A,B). International efforts should focus on closing this gap and stimulate actions for universal access to blood-derived medicine.

**Figure 2:**
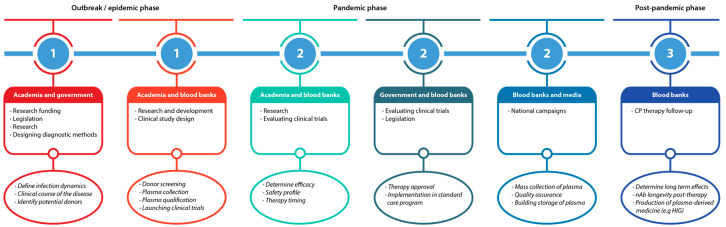
Flowchart of the pandemic preparedness program.

We have witnessed a delay in the deployment of CP therapy as a potential treatment option for COVID-19 patients. Mounting evidence derived from recent clinical trials underlines the safety and potential efficacy of this approach. The imminent threat of a second or even multiple waves of COVID-19 compels health authorities to delineate and calibrate a feasible preparedness algorithm for deploying CP as an immediate therapeutic intervention. However, the success of preparedness programs depends on the interdisciplinary actions of the scientific community, politicians, and blood transfusion authorities. Here, we will outline important aspects that should be regarded for the implementation of CP therapy in preparedness guidelines (Figure 2).

In an emerging pandemic, large-scale multicenter clinical trials are needed to evaluate the available treatment options. Valuable time was lost during the initial days of the pandemic and we must take lessons from these experiences for future emergencies. For instance, ambiguous messages concerning the COVID-19 pandemic hampered the implementation of clinical trials and patient recruitment in the US [125]. To act quickly in emergency situations, pragmatic approaches are needed for designing clinical studies and reporting results. Initially, the majority of people received CP therapy outside the context of clinical trials for compassionate use and valuable data on treatment efficacy and safety was lost. Going forward, we must rethink the traditional framework of clinical trial design to improve the efficiency of data collection and communication. How this could be improved is exemplified by the RECOVERY trial, which could serve as a landmark for large studies with multiple stakeholders [126].

Besides the clinical and scientific field, support from governmental institutes in both financial and legislative matters is imperative during the initial phases of the pandemic. From a financial perspective, governments should support the initial steps of plasma collection and laboratory testing and stimulate clinical research in the safety and efficacy of CP treatment. These efforts provide the tools for defining the dynamics of infection, clinical course of the disease and potential plasma donors. At the same time, effective steps should be taken to find the optimal titer of neutralizing antibodies. The optimal timing of CP administration and the possibility of plasma fractionations should be investigated.

Successful implementation of CP therapy heavily depends on the availability of plasma. Blood banks should aim for the acquisition of sufficient stock during the heights of the pandemic. In contrast chemically based antiviral therapies, CP is derived from human resources, which requires individuals that survived infection and acquired an adequate titer of neutralizing antibody. Therefore, the number of potential donors that mounted a robust immune response can become limited if the peak of the epidemic drops [53]. Furthermore, the immune response can be highly variable between individuals, which results in variable titers of neutralizing antibodies [19,127]. The data support the implementation of neutralizing antibody assays to determine the neutralizing capacity of the donated plasma. The timing of plasma donation could be a limiting factor since the current data demonstrate a gradual decline in neutralizing antibodies to undetectable concentrations after two to three months. To be prepared for the next waves of the epidemic, short-term collection and storage of plasma units are highly recommended. Establishing a supply of frozen CP units, with a shelf-life of 1.5 years, is the main objective of the preparedness program [54].

Besides regulatory aspects, the evolution of the virus should be tracked meticulously. For instance, the accumulation of viral mutations could negatively affect the efficacy of CP treatment [5]. Therefore, the surveillance of circulating strains and effectiveness of the stored CP units or HIG against these strains are highly recommended [128]. Since the viral genetic diversity can be different in various geographical regions, it is essential to support a local or national supply of CP [38].

The success of CP therapy fully depends on voluntary nonremunerated plasma donations of recovered patients. To inform the target population, national campaigns can be launched to increase awareness of the availability of this treatment. Different information channels, including social media, can be used to inspire grassroots engagement and participation in the donation process [129]. The motivation to donate blood could emerge from altruistic or personal reasons, to help other people or for personal credit. Especially in the latter cases, support from influencers could be an effective strategy to stimulate plasma donations. Informing the public of the curative impact of CP could spark a sense of social responsibility and inspire people to be involved in the treatment of patients.

In the upcoming months, without the availability of vaccines and curative treatments, the demand for CP and other available medicines will keep increasing. Needless to say, mass screening of (a)symptomatic individuals can guide authorities in programming their preparedness actions. Equitable access to facilities for deploying CP therapy in all countries is an imperative element for the preparedness roadmap. Critical concerns over limited resources in low- and middle-income countries should be addressed by actions of solidarity. For instance, political–economic sanctions, economic restrictions and logistic limitations should be lifted in time to make CP therapy widely available [1,130,131]. This highlights an important duty for the WHO and international collaborations.

The use of CP therapy has proven to be an effective strategy in previous epidemics. CP therapy is not only a promising approach for the transition time to develop an efficient and safe vaccine but also for the prophylactic potential after the pandemic. Importantly, it can help the healthcare workers that are exposed to infected individuals on the frontlines [53]. It is still a long way for a vaccine to arrive and mass production and distribution are important hurdles to overcome [132]. Therefore, the battle against SARS-CoV-2 could benefit from therapeutic measures like CP therapy. However, to implement this strategy successfully and to be prepared for upcoming waves, strict guidelines should be developed for the collection, testing, and transfusion of CP [133].

## 4. Conclusions

In conclusion, the implementation of CP therapy in response to an emerging pandemic requires a global effort between multiple actors from various disciplines. By learning and synchronizing our efforts in the ongoing pandemic, we can certainly turn a tragic pandemic into a beacon for the next phases of current and future pandemics.

## Figures and Tables

**Table 1:** Overview of clinical trial design and outcome for convalescent plasma (CP) therapy in COVID-19 patients.

Reference	Study Design	Time of Transfusion (Days Postadmission)	*Neutralizing Ab Titer **Anti-SARS-CoV-2 Ab Titer (Spike-Antigen Antibody)	Transfused Volume (mL/units)	Clinical Outcome	Data Collection (Days after Infusion)	Conclusion
[33]	Case series, 5 critically ill patients	10–22	*>1:40 **>1:1000	400/2	Normalizing body temperature Resolution of ARDS Decrease in SOFA Decrease/undetectable viral load Development of neutralizing Ab	12	Efficacy + no severe adverse events
[34]	Case series, 10 severely ill patients (ChiCTR2000030046)	11–20	*>1:640	200/1	Decrease/undetectable viral load, decrease in CRP Increased oxygen saturation, increased lymphocyte count, absorption of lung lesions No ARDS	3–7	Efficacy + no severe adverse events
[82]	Case series, 4 severely ill patients	12–19	**IgG titer >1:320 **IgM, OD ratio 1.22 (weakly reactive)	200–2400/1–2	Undetectable viral load Weaning from mechanical ventilation Absorption of lung lesions	11	Efficacy + no severe adverse events
[83]	Case series, 3 patients	12–27	**IgG titer >1:160	200–500/	Undetectable viral load Hospital discharge	4–26	Efficacy + anaphylactic shock in one case (plasma donor had a history of pregnancy)
[64]	Case series, 6 patients	33–50	Was not defined in the article	200–600/1–3	Development of neutralizing antibodies, resolution of consolidation		Efficacy + no severe adverse events
[73]	Case series, 1 critically ill patient	17	**IgG titer >1:320	200/1	Increased oxygen saturation Increased lymphocyte count Weaning from mechanical ventilation	11	Efficacy + no severe adverse events
[86]	Case series, 6 and 15 critically ill patients and controls, respectively	12.5	IgG-positive and IgM-negative	200–600/1	No viral shedding in most of both groups Death of 5/6 patients in the group and 14/15 in the control group	3	No severe adverse effects, CP infusion is not effective for critically ill patients at the late stages of the disease. Infusion in the early phase is recommended
[85]	Case series, 2 critically ill patient	6 and 10	IgG-positive	500/2	CRP and IL-6 normalization Decrease in viral load Resolution of lung infiltration Weaning from mechanical ventilation	24 and 26	Efficacy + no severe adverse events
[70]	Matched control study of 39 sever and life-threatening	4	**titer ≥1:320	250/2	Improvement of survival in the CP-treated group	Variable	No severe adverse effects Positive impact on survival rate
[30]	Open-label, multicenter, randomized trial, 45 severe and 58 patients with life-threatening disease (ChiCTR2000029757)	27	**<1:160 **1:160–1:1280 or >1280	4 to 13 mL/kg	No statistically significant clinical improvements 28 days post-treatment (improvements in 52% of CP recipients versus 43% of controls)	7–28	Interpretation is limited by the early termination of the trial
[31]	Case series, 25 critically ill patients	2	**1:0–1:1350	300	Resolution of ARDS Weaning from mechanical ventilation Improved clinical parameters Discharge in 20/25 patients	7–14	No severe adverse events Positive impact on survival rate
[84]	Matched control study of 316 patients with severe and life-threatening disease (NCT04554992)	3	**>1:1350 or <1:1350 (>1:150–1:1350)	300/1 or more	Weaning from mechanical ventilation Discharge from ICU to the ward Decreased ventilation time	3–28	No severe adverse events Convalescent plasma was effective in the first 72 h after admission. Here, a reduced mortality rate was observed
[81]	Open-label randomized trial with 86 patients (NCT04342182)	>4 days	*>1:80	300/1 or 2	No difference in mortality, hospital stay or disease severity was observed after 15 days	15	Prematurely stopped. At the time of inclusion, 53 of 66 patients had anti-SARS-CoV-2 antibodies at baseline
[87]	Open-label, multicenter, study with 35322 patients with severe or life-threatening (NCT04338360)	Within 3 or ≥4 days	Signal-to-cut-off (S/Co) ratio	150–250/1 or 2	7- and 30-day mortality rates were reduced in patients who received plasma with antibody titers of 1:338 or higher	7–30	Earlier time to transfusion and higher antibody levels provide signatures of efficacy. No severe adverse events
[79]	Multicenter, randomized clinical trial on 87 hospitalized patients (NCT04345523)	1	*>1:80	250–300/1	38/81 of CP recipients died or developed severe disease and required mechanical ventilation	15–29	The trial was stopped due to the drop in available patients following control of the pandemic
[80]	Open-label, phase II, multicenter, randomized controlled trial, with 464 hospitalized patients (CTRI/2020/04/024775)	Not specified	*1:20–1:1280	200/2	Resolution of dyspnea and fatigue, early clearance of viral RNA, reduce FiO^2^ requirement, weaning from mechanical ventilation CP was not associated with reduced mortality or progression to severe disease	Days 0, 1, 3, 5, 7, 14 and 28	Minimal and non-life-threatening adverse events Mortality was assessed as possibly related to CP transfusion in three patients CP therapy seemed ineffective

## References

[1] Takian A., Raoofi A., Kazempour-Ardebili S. (2020). COVID-19 battle during the toughest sanctions against Iran. Lancet.

[2] Zhou P., Yang X.-L., Wang X.-G., Hu B., Zhang L., Zhang W., Si H.-R., Zhu Y., Li B., Huang C.-L. (2020). A pneumonia outbreak associated with a new coronavirus of probable bat origin. Nature.

[3] Zhu N., Zhang D., Wang W. (2020). China Novel Coronavirus Investigating and Research Team. A novel coronavirus from patients with pneumonia in China, 2019. N. Engl. J. Med..

[4] WHO (2020). WHO made the assessment that COVID-19 can be characterized as a pandemic. WHO-Timeline COVID-19.

[5] Gasparyan A.Y., Misra D.P., Yessirkepov M., Zimba O. (2020). Perspectives of immune therapy in coronavirus disease 2019. J. Korean Med. Sci..

[6] Matthay M.A., Aldrich J.M., Gotts J.E. (2020). Treatment for severe acute respiratory distress syndrome from COVID-19. Lancet Respir. Med..

[7] Grein J., Ohmagari N., Shin D. (2020). Original: Compassionate Use of Remdesivir for Patients with Severe Covid-19. N. Engl. J. Med..

[8] Wang W., Zhang D., Du R. (2020). Original: Remdesivir in Adults with Severe Covid-19: A Randomised, Double-Blind, Placebo.

[9] Pan H., Peto R., Karim Q.A., Alejandria M., Restrepo A.M.H., Garcia C.H., Kieny M.P., Malekzadeh R., Murthy S., Preziosi M.-P. (2020). Repurposed antiviral drugs for COVID-19; interim WHO Solidarity trial results. medRxiv.

[10] Von Behring E., Kitasato S. (1991). The mechanism of diphtheria immunity and tetanus immunity in animals. 1890. Mol. Immunol..

[11] Klöppel U. (2008). Enacting Cultural Boundaries in French and German Diphtheria Serum Research. Sci. Context.

[12] Casadevall A., Scharff M.D. (1995). Return to the past: The case for antibody-based therapies in infectious diseases. Clin. Infect. Dis..

[13] Rozowski T. (1955). Emil Behring: Discoverer of antitoxins and father of serotherapy. Polski Tygodnik Lekarski.

[14] Keller M.A., Stiehm E.R. (2000). Passive immunity in prevention and treatment of infectious diseases. Clin. Microbiol. Rev..

[15] McGuire L., Redden W. (1918). The use of convalescent human serum in influenza pneumonia—A preliminary report. Am. J. Public Health.

[16] Bloch E.M., Shoham S., Casadevall A., Sachais B.S., Shaz B., Winters J.L., van Buskirk C., Grossman B.J., Joyner M., Henderson J.P. (2020). Deployment of convalescent plasma for the prevention and treatment of COVID-19. J. Clin. Investig..

[17] Lai S. (2005). Treatment of severe acute respiratory syndrome. Eur. J. Clin. Microbiol. Infect. Dis..

[18] Hung I.F., To K.K., Lee C.-K., Lee K.-L., Chan K., Yan W.-W., Liu R., Watt C.-L., Chan W.-M., Lai K.-Y. (2011). Convalescent plasma treatment reduced mortality in patients with severe pandemic influenza A (H1N1) 2009 virus infection. Clin. Infect. Dis..

[19] Arabi Y.M., Hajeer A.H., Luke T., Raviprakash K., Balkhy H., Johani S., Al-Dawood A., Al-Qahtani S., Al-Omari A., Al-Hameed F. (2016). Feasibility of using convalescent plasma immunotherapy for MERS-CoV infection, Saudi Arabia. Emerg. Infect. Dis..

[20] WHO (2014). Use of Convalescent Whole Blood or Plasma Collected from Patients Recovered from Ebola Virus Disease for Transfusion, as an Empirical Treatment During Outbreaks: Interim Guidance for National Health Authorities and Blood Transfusion Services.

[21] Sahr F., Ansumana R., Massaquoi T., Idriss B., Sesay F., Lamin J., Baker S., Nicol S., Conton B., Johnson W. (2017). Evaluation of convalescent whole blood for treating Ebola Virus Disease in Freetown, Sierra Leone. J. Infect..

[22] Zhang L., Liu Y. (2020). Potential interventions for novel coronavirus in China: A systematic review. J. Med. Virol..

[23] Mair-Jenkins J., Saavedra-Campos M., Baillie J.K., Cleary P., Khaw F.-M., Lim W.S., Makki S., Rooney K.D., Group C.P.S., Nguyen-Van-Tam J.S. (2015). The effectiveness of convalescent plasma and hyperimmune immunoglobulin for the treatment of severe acute respiratory infections of viral etiology: A systematic review and exploratory meta-analysis. J. Infect. Dis..

[24] Luke T.C., Kilbane E.M., Jackson J.L., Hoffman S.L. (2006). Meta-analysis: Convalescent blood products for Spanish influenza pneumonia: A future H5N1 treatment?. Ann. Intern. Med..

[25] Rajendran K., Narayanasamy K., Rangarajan J., Rathinam J., Natarajan M., Ramachandran A. (2020). Convalescent plasma transfusion for the treatment of COVID-19: Systematic review. J. Med. Virol..

[26] Cheng Y., Wong R., Soo Y., Wong W., Lee C., Ng M., Chan P., Wong K., Leung C., Cheng G. (2005). Use of convalescent plasma therapy in SARS patients in Hong Kong. Eur. J. Clin. Microbiol. Infect. Dis..

[27] Arabi Y., Balkhy H., Hajeer A.H., Bouchama A., Hayden F.G., Al-Omari A., Al-Hameed F.M., Taha Y., Shindo N., Whitehead J. (2015). Feasibility, safety, clinical, and laboratory effects of convalescent plasma therapy for patients with Middle East respiratory syndrome coronavirus infection: A study protocol. SpringerPlus.

[28] Chen L., Xiong J., Bao L., Shi Y. (2020). Convalescent plasma as a potential therapy for COVID-19. Lancet Infect. Dis..

[29] Joyner M.J., Wright R.S., Fairweather D., Senefeld J.W., Bruno K.A., Klassen S.A., Carter R.E., Klompas A.M., Wiggins C.C., Shepherd J.R. (2020). Early safety indicators of COVID-19 convalescent plasma in 5000 patients. J. Clin. Investig..

[30] Li L., Zhang W., Hu Y., Tong X., Zheng S., Yang J., Kong Y., Ren L., Wei Q., Mei H. (2020). Effect of Convalescent Plasma Therapy on Time to Clinical Improvement in Patients with Severe and Life-threatening COVID-19: A Randomized Clinical Trial. JAMA.

[31] Salazar E., Perez K.K., Ashraf M., Chen J., Castillo B., Christensen P.A., Eubank T., Bernard D.W., Eagar T.N., Long S.W. (2020). Treatment of COVID-19 Patients with Convalescent Plasma. Am. J. Pathol..

[32] Cortegiani A., Ingoglia G., Ippolito M., Giarratano A., Einav S. (2020). A systematic review on the efficacy and safety of chloroquine for the treatment of COVID-19. J. Crit. Care.

[33] Shen C., Wang Z., Zhao F., Yang Y., Li J., Yuan J., Wang F., Li D., Yang M., Xing L. (2020). Treatment of 5 critically ill patients with COVID-19 with convalescent plasma. JAMA.

[34] Duan K., Liu B., Li C., Zhang H., Yu T., Qu J., Zhou M., Chen L., Meng S., Hu Y. (2020). Effectiveness of convalescent plasma therapy in severe COVID-19 patients. Proc. Natl. Acad. Sci. USA.

[35] Zhang W., Du R.-H., Li B., Zheng X.-S., Yang X.-L., Hu B., Wang Y.-Y., Xiao G.-F., Yan B., Shi Z.-L. (2020). Molecular and serological investigation of 2019-nCoV infected patients: Implication of multiple shedding routes. Emerg. Microbes Infect..

[36] Tanne J.H. (2020). Covid-19: FDA approves use of convalescent plasma to treat critically ill patients. BMJ.

[37] FDA Recommendations for Investigational COVID-19 Convalescent Plasma. https://www.fda.gov/vaccines-blood-biologics/investigational-new-drug-ind-or-device-exemption-ide-process-cber/recommendations-investigational-covid-19-convalescent-plasma.

[38] Perotti C., Del Fante C., Baldanti F., Franchini M., Percivalle E., Vecchio Nepita E., Seminari E., De Silvestri A., Bruno R., Klersy C. (2020). Plasma from donors recovered from the new Coronavirus 2019 as therapy for critical patients with COVID-19 (COVID-19 plasma study): A multicentre study protocol. Intern. Emerg Med..

[39] Haematology T.L. (2020). The resurgence of convalescent plasma therapy. Lancet. Haematol..

[40] Langhi D.M., Junior G.C.D.S., Bordin J.O. (2020). COVID-19 convalescent plasma transfusion. Hematol. Transfus. Cell Ther..

[41] Tiberghien P., de Lamballerie X., Morel P., Gallian P., Lacombe K., Yazdanpanah Y. (2020). Collecting and evaluating convalescent plasma for COVID-19 treatment: Why and how?. Vox Sang..

[42] Syal K. (2020). COVID-19: Herd immunity and convalescent plasma transfer therapy. J. Med. Virol..

[43] Franchini M., Marano G., Velati C., Pati I., Pupella S., Liumbruno G.M. (2020). Operational protocol for donation of anti-COVID-19 convalescent plasma in Italy. Vox Sang..

[44] Epstein J., Burnouf T. (2020). Points to consider in the preparation and transfusion of COVID-19 convalescent plasma. Vox Sang..

[45] Xie L., Liu Y., Fan B., Xiao Y., Tian Q., Chen L., Zhao H., Chen W. (2005). Dynamic changes of serum SARS-coronavirus IgG, pulmonary function and radiography in patients recovering from SARS after hospital discharge. Respir. Res..

[46] Liu W., Fontanet A., Zhang P.-H., Zhan L., Xin Z.-T., Baril L., Tang F., Lv H., Cao W.-C. (2006). Two-year prospective study of the humoral immune response of patients with severe acute respiratory syndrome. J. Infect. Dis..

[47] Haveri A., Smura T., Kuivanen S., Österlund P., Hepojoki J., Ikonen N., Pitkäpaasi M., Blomqvist S., Rönkkö E., Kantele A. (2020). Serological and molecular findings during SARS-CoV-2 infection: The first case study in Finland, January to February 2020. Eurosurveillance.

[48] Muruato A.E., Fontes-Garfias C.R., Ren P., Garcia-Blanco M.A., Menachery V.D., Xie X., Shi P.-Y. (2020). A high-throughput neutralizing antibody assay for COVID-19 diagnosis and vaccine evaluation. bioRxiv.

[49] Wu F., Wang A., Liu M., Wang Q., Chen J., Xia S., Ling Y., Zhang Y., Xun J., Lu L. (2020). Neutralizing antibody responses to SARS-CoV-2 in a COVID-19 recovered patient cohort and their implications. medRxiv.

[50] Brown J.F., Dye J.M., Tozay S., Jeh-Mulbah G., Wohl D.A., Fischer W.A., Cunningham C.K., Rowe K., Zacharias P., van Hasselt J. (2018). Anti–Ebola Virus Antibody Levels in Convalescent Plasma and Viral Load After Plasma Infusion in Patients With Ebola Virus Disease. J. Infect. Dis..

[51] Amanat F., Stadlbauer D., Strohmeier S., Nguyen T.H., Chromikova V., McMahon M., Jiang K., Arunkumar G.A., Jurczyszak D., Polanco J. (2020). A serological assay to detect SARS-CoV-2 seroconversion in humans. Nat. Med..

[52] Wong H.K., Lee C.K., Hung I.F., Leung J.N., Hong J., Yuen K.Y., Lin C.K. (2010). Practical limitations of convalescent plasma collection: A case scenario in pandemic preparation for influenza A (H1N1) infection. Transfusion.

[53] Sullivan H.C., Roback J.D. (2020). Convalescent plasma: Therapeutic hope or hopeless strategy in the SARS-CoV-2 pandemic. Transfus. Med. Rev..

[54] Long Q.-X., Tang X.-J., Shi Q.-L., Li Q., Deng H.-J., Yuan J., Hu J.-L., Xu W., Zhang Y., Lv F.-J. (2020). Clinical and immunological assessment of asymptomatic SARS-CoV-2 infections. Nat. Med..

[55] Sekine T., Perez-Potti A., Rivera-Ballesteros O., Straling K., Gorin J.-B., Olsson A., Llewellyn-Lacey S., Kamal H., Bogdanovic G., Muschiol S. (2020). Robust T cell immunity in convalescent individuals with asymptomatic or mild COVID-19. BioRXiv.

[56] Li L., Tong X., Chen H., He R., Lv Q., Yang R., Zhao L., Wang J., Xu H., Liu C. (2020). Characteristics and serological patterns of COVID-19 convalescent plasma donors: Optimal donors and timing of donation. Transfusion.

[57] Wong H.-K., Lee C.-K. (2020). Pivotal role of convalescent plasma in managing emerging infectious diseases. Vox Sang..

[58] Zhao J., Yuan Q., Wang H., Liu W., Liao X., Su Y., Wang X., Yuan J., Li T., Li J. (2020). Antibody responses to SARS-CoV-2 in patients of novel coronavirus disease 2019. Clin. Infect. Dis..

[59] Okba N.M., Müller M.A., Li W., Wang C., GeurtsvanKessel C.H., Corman V.M., Lamers M.M., Sikkema R.S., de Bruin E., Chandler F.D. (2020). Severe acute respiratory syndrome coronavirus 2-specific antibody responses in coronavirus disease 2019 patients. Emerg. Infect. Dis..

[60] Guo L., Ren L., Yang S., Xiao M., Chang D., Yang F., Dela Cruz C.S., Wang Y., Wu C., Xiao Y. (2020). Profiling early humoral response to diagnose novel coronavirus disease (COVID-19). Clin. Infect. Dis..

[61] Bullard J., Dust K., Funk D., Strong J.E., Alexander D., Garnett L., Boodman C., Bello A., Hedley A., Schiffman Z. (2020). Predicting infectious SARS-CoV-2 from diagnostic samples. Clin. Infect. Dis..

[62] Wang M., Wu Q., Xu W., Qiao B., Wang J., Zheng H., Jiang S., Mei J., Wu Z., Deng Y. (2020). Clinical diagnosis of 8274 samples with 2019-novel coronavirus in Wuhan. medRxiv.

[63] Diao B., Wen K., Zhang J., Chen J., Han C., Chen Y., Wang S., Deng G., Zhou H., Wu Y. (2020). Accuracy of a nucleocapsid protein antigen rapid test in the diagnosis of SARS-CoV-2 infection. Clin. Microbiol. Infect..

[64] Ye M., Fu D., Ren Y., Wang F., Wang D., Zhang F., Xia X., Lv T. (2020). Treatment with convalescent plasma for COVID-19 patients in Wuhan, China. J. Med. Virol..

[65] Huang C., Wang Y., Li X., Ren L., Zhao J., Hu Y., Zhang L., Fan G., Xu J., Gu X. (2020). Clinical features of patients infected with 2019 novel coronavirus in Wuhan, China. Lancet.

[66] Yang Y., Yang M., Shen C., Wang F., Yuan J., Li J., Zhang M., Wang Z., Xing L., Wei J. (2020). Laboratory diagnosis and monitoring the viral shedding of 2019-nCoV infections. MedRxiv.

[67] Chan J.F.-W., Yuan S., Kok K.-H., To K.K.-W., Chu H., Yang J., Xing F., Liu J., Yip C.C.-Y., Poon R.W.-S. (2020). A familial cluster of pneumonia associated with the 2019 novel coronavirus indicating person-to-person transmission: A study of a family cluster. Lancet.

[68] Lamers M.M., Beumer J., van der Vaart J., Knoops K., Puschhof J., Breugem T.I., Ravelli R.B., van Schayck J.P., Mykytyn A.Z., Duimel H.Q. (2020). SARS-CoV-2 productively infects human gut enterocytes. Science.

[69] Jeong H.W., Kim S.-M., Kim H.-S., Kim Y.-I., Kim J.H., Cho J.Y., Kim S.-h., Kang H., Kim S.-G., Park S.-J. (2020). Viable SARS-CoV-2 in various specimens from COVID-19 patients. Clin. Microbiol. Infect..

[70] Liu S.T., Lin H.-M., Baine I., Wajnberg A., Gumprecht J.P., Rahman F., Rodriguez D., Tandon P., Bassily-Marcus A., Bander J. (2020). Convalescent plasma treatment of severe COVID-19: A matched control study. medRxiv.

[71] Burnouf T., Dye J.M., Abayomi A. (2017). Convalescent plasma and the dose of Ebola virus antibodies. N. Engl. J. Med..

[72] Meyer B., Reimerink J., Torriani G., Brouwer F., Godeke G.-J., Yerly S., Hoogerwerf M., Vuilleumier N., Kaiser L., Eckerle I. (2020). Validation and clinical evaluation of a SARS-CoV-2 Surrogate Virus Neutralisation Test (sVNT). Emerg. Microbes Infect..

[73] Zhang L., Pang R., Xue X., Bao J., Ye S., Dai Y., Zheng Y., Fu Q., Hu Z., Yi Y. (2020). Anti-SARS-CoV-2 virus antibody levels in convalescent plasma of six donors who have recovered from COVID-19. Aging.

[74] Ye G., Pan Z., Pan Y., Deng Q., Chen L., Li J., Li Y., Wang X. (2020). Clinical characteristics of severe acute respiratory syndrome coronavirus 2 reactivation. J. Infect..

[75] Mallapaty S. (2020). Will antibody tests for the coronavirus really change everything?. Nature.

[76] Okba N.M., Muller M.A., Li W., Wang C., GeurtsvanKessel C.H., Corman V.M., Lamers M.M., Sikkema R.S., de Bruin E., Chandler F.D. (2020). SARS-CoV-2 specific antibody responses in COVID-19 patients. medRxiv.

[77] To K.K.-W., Hung I.F.-N., Ip J.D., Chu A.W.-H., Chan W.-M., Tam A.R., Fong C.H.-Y., Yuan S., Tsoi H.-W., Ng A.C.-K. (2020). COVID-19 re-infection by a phylogenetically distinct SARS-coronavirus-2 strain confirmed by whole genome sequencing. Clin. Infect. Dis..

[78] Huang A.T., Garcia-Carreras B., Hitchings M.D., Yang B., Katzelnick L.C., Rattigan S.M., Borgert B.A., Moreno C.A., Solomon B.D., Rodriguez-Barraquer I. (2020). A systematic review of antibody mediated immunity to coronaviruses: Antibody kinetics, correlates of protection, and association of antibody responses with severity of disease. medRxiv.

[79] Avendano-Sola C., Ramos-Martinez A., Munez-Rubio E., Ruiz-Antoran B., de Molina R.M., Torres F., Fernandez-Cruz A., Callejas-Diaz A., Calderon J., Payares-Herrera C. (2020). Convalescent Plasma for COVID-19: A multicenter, randomized clinical trial. medRxiv.

[80] Agarwal A., Mukherjee A., Kumar G., Chatterjee P., Bhatnagar T., Malhotra P., Latha B., Bundas S., Kumar V., Dosi R. (2020). Convalescent plasma in the management of moderate COVID-19 in India: An open-label parallel-arm phase II multicentre randomized controlled trial (PLACID Trial). MedRxiv.

[81] Gharbharan A., Jordans C.C., GeurtsvanKessel C., den Hollander J.G., Karim F., Mollema F.P., Stalenhoef J.E., Dofferhoff A., Ludwig I., Koster A. (2020). Convalescent Plasma for COVID-19. A randomized clinical trial. MedRxiv.

[82] Zhang B., Liu S., Tan T., Huang W., Dong Y., Chen L., Chen Q., Zhang L., Zhong Q., Zhang X. (2020). Treatment with convalescent plasma for critically ill patients with SARS-CoV-2 infection. Chest.

[83] Pei S., Yuan X., Zhang Z.Z., Yao R.R., Xie Y., Shen M.M., Li B.B., Chen X., Yin M. (2020). Convalescent plasma to treat covid-19: Chinese strategy and experiences. medRxiv.

[84] Salazar E., Christensen P.A., Graviss E.A., Nguyen D.T., Castillo B., Chen J., Lopez B.V., Eagar T.N., Yi X., Zhao P. (2020). Treatment of coronavirus disease 2019 patients with convalescent plasma reveals a signal of significantly decreased mortality. Am. J. Pathol..

[85] Ahn J.Y., Sohn Y., Lee S.H., Cho Y., Hyun J.H., Baek Y.J., Jeong S.J., Kim J.H., Ku N.S., Yeom J.-S. (2020). Use of convalescent plasma therapy in two COVID-19 patients with acute respiratory distress syndrome in Korea. J. Korean Med. Sci..

[86] Zeng Q.-L., Yu Z.-J., Gou J.-J., Li G.-M., Ma S.-H., Zhang G.-F., Xu J.-H., Lin W.-B., Cui G.-L., Zhang M.-M. (2020). Effect of convalescent plasma therapy on viral shedding and survival in patients with coronavirus disease 2019. J. Infect. Dis..

[87] Joyner M.J., Senefeld J.W., Klassen S.A., Mills J.R., Johnson P.W., Theel E.S., Wiggins C.C., Bruno K.A., Klompas A.M., Lesser E.R. (2020). Effect of convalescent plasma on mortality among hospitalized patients with COVID-19: Initial three-month experience. medrxiv.

[88] Casadevall A., Joyner M.J., Pirofski L.-A. (2020). A Randomized Trial of Convalescent Plasma for COVID-19—Potentially Hopeful Signals. JAMA.

[89] Morabito C.J., Gangadharan B. (2020). Active Therapy with Passive Immunotherapy May Be Effective in the Fight against Covid-19. Clin. Transl. Sci..

[90] Rubin R. (2020). Testing an old therapy against a new disease: Convalescent plasma for COVID-19. JAMA.

[91] Yeh K.-M., Chiueh T.-S., Siu L., Lin J.-C., Chan P.K., Peng M.-Y., Wan H.-L., Chen J.-H., Hu B.-S., Perng C.-L. (2005). Experience of using convalescent plasma for severe acute respiratory syndrome among healthcare workers in a Taiwan hospital. J. Antimicrob. Chemother..

[92] Zhou B., Zhong N., Guan Y. (2007). Treatment with convalescent plasma for influenza A (H5N1) infection. N. Engl. J. Med..

[93] Burnouf T., Radosevich M. (2003). Treatment of severe acute respiratory syndrome with convalescent plasma. Hong Kong Med. J..

[94] Garcia P.D.W., Fumeaux T., Guerci P., Heuberger D.M., Montomoli J., Roche-Campo F., Schuepbach R.A., Hilty M.P., RISC-19-ICU Investigators (2020). Prognostic factors associated with mortality risk and disease progression in 639 critically ill patients with COVID-19 in Europe: Initial report of the international RISC-19-ICU prospective observational cohort. EClinicalMedicine.

[95] Richardson S., Hirsch J.S., Narasimhan M., Crawford J.M., McGinn T., Davidson K.W., Barnaby D.P., Becker L.B., Chelico J.D., Cohen S.L. (2020). Presenting characteristics, comorbidities, and outcomes among 5700 patients hospitalized with COVID-19 in the New York City area. JAMA.

[96] Wang K., Zhang Z., Yu M., Tao Y., Xie M. (2020). 15-day mortality and associated risk factors for hospitalized patients with COVID-19 in Wuhan, China: An ambispective observational cohort study. Intensive Care Med..

[97] Nurtop E., Villarroel P.M.S., Pastorino B., Ninove L., Drexler J.F., Roca Y., Gake B., Dubot-Peres A., Grard G., Peyrefitte C. (2018). Combination of ELISA screening and seroneutralisation tests to expedite Zika virus seroprevalence studies. Virol. J..

[98] Eickmann M., Gravemann U., Handke W., Tolksdorf F., Reichenberg S., Müller T.H., Seltsam A. (2018). Inactivation of Ebola virus and Middle East respiratory syndrome coronavirus in platelet concentrates and plasma by ultraviolet C light and methylene blue plus visible light, respectively. Transfusion.

[99] Faddy H.M., Fryk J.J., Hall R.A., Young P.R., Reichenberg S., Tolksdorf F., Sumian C., Gravemann U., Seltsam A., Marks D.C. (2019). Inactivation of yellow fever virus in plasma after treatment with methylene blue and visible light and in platelet concentrates following treatment with ultraviolet C light. Transfusion.

[100] Ragan I., Hartson L., Pidcoke H., Bowen R., Goodrich R. (2020). Pathogen reduction of SARS-CoV-2 virus in plasma and whole blood using riboflavin and UV light. PLoS ONE.

[101] Chang L., Yan Y., Wang L. (2020). Coronavirus disease 2019: Coronaviruses and blood safety. Transfus. Med. Rev..

[102] Rodriguez-Morales A.J., Cardona-Ospina J.A., Gutiérrez-Ocampo E., Villamizar-Peña R., Holguin-Rivera Y., Escalera-Antezana J.P., Alvarado-Arnez L.E., Bonilla-Aldana D.K., Franco-Paredes C., Henao-Martinez A.F. (2020). Clinical, laboratory and imaging features of COVID-19: A systematic review and meta-analysis. Travel Med. Infect. Dis..

[103] Benson A.B., Moss M., Silliman C.C. (2009). Transfusion-related acute lung injury (TRALI): A clinical review with emphasis on the critically ill. Br. J. Haematol..

[104] Ooley P. (2017). AABB Standards for Blood Banks and Transfusion Services.

[105] Mora-Rillo M., Arsuaga M., Ramírez-Olivencia G., de la Calle F., Borobia A.M., Sánchez-Seco P., Lago M., Figueira J.C., Fernández-Puntero B., Viejo A. (2015). Acute respiratory distress syndrome after convalescent plasma use: Treatment of a patient with Ebola virus disease contracted in Madrid, Spain. Lancet Respir. Med..

[106] Llitjos J.F., Leclerc M., Chochois C., Monsallier J.M., Ramakers M., Auvray M., Merouani K. (2020). High incidence of venous thromboembolic events in anticoagulated severe COVID-19 patients. J. Thromb. Haemost..

[107] Flipse J., Diosa-Toro M.A., Hoornweg T.E., Van De Pol D.P., Urcuqui-Inchima S., Smit J.M. (2016). Antibody-dependent enhancement of dengue virus infection in primary human macrophages; balancing higher fusion against antiviral responses. Sci. Rep..

[108] Liu L., Wei Q., Lin Q., Fang J., Wang H., Kwok H., Tang H., Nishiura K., Peng J., Tan Z. (2019). Anti–spike IgG causes severe acute lung injury by skewing macrophage responses during acute SARS-CoV infection. JCI Insight.

[109] Tirado S.M.C., Yoon K.-J. (2003). Antibody-dependent enhancement of virus infection and disease. Viral Immunol..

[110] Wan Y., Shang J., Sun S., Tai W., Chen J., Geng Q., He L., Chen Y., Wu J., Shi Z. (2020). Molecular mechanism for antibody-dependent enhancement of coronavirus entry. J. Virol..

[111] Katzelnick L.C., Gresh L., Halloran M.E., Mercado J.C., Kuan G., Gordon A., Balmaseda A., Harris E. (2017). Antibody-dependent enhancement of severe dengue disease in humans. Science.

[112] Robinson Jr W.E., Montefiori D.C., Gillespie D.H., Mitchell W.M. (1989). Complement-mediated, antibody-dependent enhancement of HIV-1 infection in vitro is characterized by increased protein and RNA syntheses and infectious virus release. J. Acquir. Immune Defic. Syndr..

[113] Uhr J.W., Baumann J.B. (1961). Antibody formation: I. The suppression of antibody formation by passively administered antibody. J. Exp. Med..

[114] Hung I.F., To K.K., Lee C.-K., Lee K.-L., Yan W.-W., Chan K., Chan W.-M., Ngai C.-W., Law K.-I., Chow F.-L. (2013). Hyperimmune IV immunoglobulin treatment: A multicenter double-blind randomized controlled trial for patients with severe 2009 influenza A (H1N1) infection. Chest.

[115] Traggiai E., Becker S., Subbarao K., Kolesnikova L., Uematsu Y., Gismondo M.R., Murphy B.R., Rappuoli R., Lanzavecchia A. (2004). An efficient method to make human monoclonal antibodies from memory B cells: Potent neutralization of SARS coronavirus. Nat. Med..

[116] Corti D., Zhao J., Pedotti M., Simonelli L., Agnihothram S., Fett C., Fernandez-Rodriguez B., Foglierini M., Agatic G., Vanzetta F. (2015). Prophylactic and postexposure efficacy of a potent human monoclonal antibody against MERS coronavirus. Proc. Natl. Acad. Sci. USA.

[117] Corti D., Misasi J., Mulangu S., Stanley D.A., Kanekiyo M., Wollen S., Ploquin A., Doria-Rose N.A., Staupe R.P., Bailey M. (2016). Protective monotherapy against lethal Ebola virus infection by a potently neutralizing antibody. Science.

[118] Levine M.M. (2019). Monoclonal Antibody Therapy for Ebola Virus Disease.

[119] Regeneron Regeneron’s Regn-Cov2 Antibody Cocktail Reduced Viral Levels and Improved Symptoms in Non-Hospitalized Covid-19 Patients. https://investor.regeneron.com/news-releases/news-release-details/regenerons-regn-cov2-antibody-cocktail-reduced-viral-levels-and/.

[120] Cohen J. The Race Is on for Antibodies That Stop the New Coronavirus. https://www.sciencemag.org/news/2020/05/race-antibodies-stop-new-coronavirus.

[121] Company ELA (2020). Lilly Statement on the NIAID Decision to Pause Enrollment in ACTIV-3 Clinical Trial.

[122] Cao Y., Su B., Guo X., Sun W., Deng Y., Bao L., Zhu Q., Zhang X., Zheng Y., Geng C. (2020). Potent neutralizing antibodies against SARS-CoV-2 identified by high-throughput single-cell sequencing of convalescent patients’ B cells. Cell.

[123] Shanmugaraj B., Siriwattananon K., Wangkanont K., Phoolcharoen W. (2020). Perspectives on monoclonal antibody therapy as potential therapeutic intervention for Coronavirus disease-19 (COVID-19). Asian Pac. J. Allergy Immunol..

[124] Tian X., Li C., Huang A., Xia S., Lu S., Shi Z., Lu L., Jiang S., Yang Z., Wu Y. (2020). Potent binding of 2019 novel coronavirus spike protein by a SARS coronavirus-specific human monoclonal antibody. Emerg. Microbes Infect..

[125] Burki T.K. (2020). Completion of clinical trials in light of COVID-19. Lancet. Respir. Med..

[126] Wise J., Coombes R. (2020). Covid-19: The inside story of the RECOVERY trial. BMJ.

[127] Park W.B., Perera R.A., Choe P.G., Lau E.H., Choi S.J., Chun J.Y., Oh H.S., Song K.-H., Bang J.H., Kim E.S. (2015). Kinetics of serologic responses to MERS coronavirus infection in humans, South Korea. Emerg. Infect. Dis..

[128] Roback J.D., Guarner J. (2020). Convalescent plasma to treat COVID-19: Possibilities and challenges. JAMA.

[129] Pourkarim M., Van Espen L., Thijssen M., Van Ranst M., Pourkarim M. (2018). How adequate social media management supports the viral Hepatitis elimination program. Hepat. Mon..

[130] Pourkarim M.R., Razavi H., Lemey P., Van Ranst M. (2018). Iran’s hepatitis elimination programme is under threat. Lancet.

[131] Pourkarim M.R., Thijssen M., Alavian S.M., Van Ranst M. (2019). Natural disasters pose a challenge for hepatitis elimination in Iran. Lancet. Gastroenterol. Hepatol..

[132] Kissler S.M., Tedijanto C., Goldstein E., Grad Y.H., Lipsitch M. (2020). Projecting the transmission dynamics of SARS-CoV-2 through the postpandemic period. Science.

[133] Hall M.A., Studdert D.M. (2020). Privileges and immunity certification during the COVID-19 pandemic. JAMA.

